# Brassinosteroids alleviate high-temperature injury in *Ficus concinna* seedlings via maintaining higher antioxidant defence and glyoxalase systems

**DOI:** 10.1093/aobpla/plv009

**Published:** 2015-01-21

**Authors:** Song Heng Jin, Xue Qin Li, G. Geoff Wang, Xiang Tao Zhu

**Affiliations:** 1 Jiyang College, Zhejiang A & F University, Zhuji, Zhejiang Province 311800, China; 2 School of Forestry and Biotechnology, Zhejiang A & F University, Lin'an, Zhejiang Province 311300, China; 3 Department of Forestry and Natural Resources, Clemson University, 226 Lehotsky Hall, Clemson, SC 29634-0317, USA

**Keywords:** Antioxidants, brassinosteroids, glyoxalase system, high-temperature stress, methylglyoxal

## Abstract

Although brassinosteroids (BRs) play crucial roles in plant development and stress tolerance, the mechanisms by which they have these effects are poorly understood. Here, we investigated the possible mechanism of exogenously applied BRs on reactive oxygen species (ROS), antioxidant defence and methylglyoxal (MG) detoxification systems in *Ficus concinna* seedlings grown under high-temperature (HT) stress for 48 h. Our results showed that the activities of ascorbate peroxidase (APX), superoxide dismutase (SOD), glutathione *S*-transferase (GST), glutathione peroxidase (GPX) and glyoxalase II (Gly II) were increased under two levels of HT stress. Compared with control the activities of catalase (CAT) and dehydroascorbate reductase (DHAR) were not changed due to HT stress. The activities of glutathione reductase (GR), monodehydroascorbate reductase (MDHAR) and glyoxalase I (Gly I) were increased only at moderate HT stress. Despite these protective mechanisms, HT stress induced oxidative stress in *F. concinna* seedlings, as indicated by the increased levels of ROS, malondialdehyde (MDA) and MG, and the reductions in chlorophyll levels and relative water content. The contents of reduced glutathione (GSH) and ascorbate (AsA) were not changed under moderate HT stress. Spraying with 24-epibrassinolide (EBR) alone had little influence on the non-enzymatic antioxidants and the activities of antioxidant enzymes. However, EBR pretreatment under HT stress resulted in an increase in GSH and AsA content, maintenance of high redox state of GSH and AsA, and enhanced ROS and MG detoxification by further elevating the activities of SOD, GST, GPX, APX, MDHAR, GR, DHAR, Gly I and Gly II, as evident by lower level of ROS, MDA and MG. It may be concluded that EBR could alleviate the HT-induced oxidative stress by increasing the enzymatic and non-enzymatic antioxidant defence, and glyoxalase systems in *F. concinna* seedlings.

## Abstract (precis)

Brassinosteroids play a significant role in the amelioration of various biotic and abiotic stresses. To investigate the effects of exogenously applied brassinosteroids on the thermotolerance of plants, reactive oxygen species (ROS), antioxidant defense and methylglyoxal (MG) detoxification systems were examined in *Ficus concinna* seedlings with or without 24-epibrassinolide (EBR) application. Our results showed that EBR treatment reduced high temperature-induced increases in the levels of ROS, MG and lipid peroxidation. We also demonstrate that EBR attenuates high temperature-induced oxidative stress by simultaneously increasing non-enzymatic and enzymatic antioxidant responses, as well as MG detoxification systems.

## Introduction

Climatic models predict that greenhouse gases will gradually increase the world's average ambient temperature (Sharkey 2005). Hence, plants growing in temperate climates will more often be exposed to high-temperature (HT) stress conditions, adversely affecting plant growth and survival in a number of ways. High temperature is known to affect membrane-linked processes due to alteration in membrane fluidity and permeability (Wahid *et al*. 2007). High temperature stress may scorch leaves, delay seed germination, disrupt photosynthesis and respiration systems, retard growth, damage plant organs and eventually lead to plant death (Guilioni *et al*. 1997; Wahid *et al*. 2007; Hasanuzzaman *et al*. 2013). High temperature-induced limitation in photosynthesis causes light energy absorbed by photosystem antennae in excess of what is required to drive photosynthetic CO_2_ assimilation (Jin *et al*. 2010). The excess energy accumulated in plant cells results in producing large amounts of reactive oxygen species (ROS) including superoxide $({{\text{O}}_{2}}^{∙-}),$ hydrogen peroxide (H_2_O_2_), singlet oxygen (^1^O_2_) and hydroxyl radicals (•OH), which all have greater toxicity potentials on biomolecules and biomembranes in plants (Yin *et al*. 2008; Hasanuzzaman *et al*. 2013). In addition to the generation of ROS, accumulation of methylglyoxal (MG), a toxic compound, has been reported under various extreme environmental stress condition and its detoxification might be a strategy for tolerance against various abiotic stresses (Yadav *et al*. 2005*a*, *b*; Hasanuzzaman *et al*. 2011*a*, *b*). Disruption of these protective mechanisms can cause oxidative stress, leading to oxidative damage.

Previous studies have shown that plants have developed a series of both enzymatic and non-enzymatic detoxification systems to counteract ROS, thereby protecting cells from oxidative damage (Apel and Hirt 2004). These enzymatic antioxidant systems include superoxide dismutase (SOD), peroxidase (POD), catalase (CAT), ascorbate peroxidase (APX) monodehydroascorbate reductase (MDHAR), dehydroascorbate reductase (DHAR), glutathione reductase (GR), glutathione peroxidase (GPX) and glutathione *S*-transferase (GST) (Apel and Hirt 2004). Non-enzymatic antioxidants include ascorbate (AsA), reduced glutathione (GSH), tocopherol, flavonoids, alkaloids, carotenoids and anthocyanins (Blokhina *et al*. 2003; Apel and Hirt 2004). Likewise, plant cells also possess an MG detoxifying glyoxalase system comprising two enzymes; glyoxalase I (Gly I) and glyoxalase II (Gly II). Gly I utilizes GSH to convert MG into its thioester, whereas the Gly II hydrolyzes this thioester to regenerate GSH (Yadav *et al*. 2005*a*, *b*). Therefore, antioxidant resistance mechanisms may provide a strategy to enhance heat tolerance, and processes underlying antioxidant responses to HT stress must be clearly understood.

*Ficus concinna* var. *subsessilis* is a very important tree species in the tropical and subtropical area, and it is mainly distributed in Southeast Asia and the south of China. High temperature prevailing at the sowing time often imposes severe limitation on early germination pattern and subsequent seedling establishment of *F. concinna* (Jin *et al*. 2009). To better understand plant responses to HT stress, it is necessary to identify the mechanisms involved in protection during HT stress. Brassinosteroids (BRs), a recent class of plant hormone, play a critical role in various physiological and biochemical processes in plants, like stem elongation, pollen tube growth, vascular differentiation, leaf bending and epinasty, root inhibition, induction of ethylene biosynthesis, proton pump activation, regulation of gene expression, nucleic acid and protein synthesis and photosynthesis (Clouse and Sasse 1998; Steber and McCourt 2001; Sasse 2003; Yu *et al*. 2004; Pereira-Netto *et al*. 2006, 2009). In addition to their role in plant development, BRs have been reported to provide protection to plant from various environmental stresses, including heat (Dhaubhadel *et al*. 1999, 2002; Mazorra *et al*. 2002; Bajguz 2009), salinity (Ali *et al*. 2008; Hayat *et al*. 2010), drought (Yuan *et al*. 2010; Li *et al*. 2012), chilling (Huang *et al*. 2006; Qu *et al*. 2011) and metal stress (Hayat *et al*. 2007; Ali *et al*. 2008; Choudhary *et al*. 2010). One mechanism that may be involved in resistance to many types of stresses by escalating activity of enzymes involved in antioxidant defence system. For instance, BRs increased the activity of CAT, peroxidase and SOD in responding to HT in tomato leaves (Mazorra *et al*. 2002). Numerous recent studies have claimed that efficient induction of ascorbate–glutathione and glyoxalase systems using exogenous protectants such as nitric acid, salicylic acid and selenium could improve the resistance to various stresses (Hasanuzzaman *et al*. 2011*a*, *b*, 2012*a*, *b*; Hasanuzzaman and Fujita 2013; Mostofa and Fujita 2013). However, there is little evidence for the involvement of BRs in the regulation of ascorbate–glutathione and glyoxalase systems in higher plants. The mechanism by which HT stress affects ascorbate–glutathione and glyoxalase systems also remains unclear. Thus, the present study aimed to evaluate the effect of exogenous BRs on the activity of the antioxidant and glyoxalase pathway enzymes under HT stress. To our knowledge, currently there is no information available on the possible beneficial effects of exogenous application of BRs on MG detoxification system in plants grown under HT stress.

## Methods

### Plant materials and stress treatments

Two-year-old seedlings of *F. concinna* var. *subsessilis* were obtained from Zhejiang Subtropical Crop Institute in Wenzhou, China. The seedlings were transplanted to plastic tubes (20.5 cm tall, 18 cm top diameter) filled with a 1 : 1 (v/v) mixture of coarse sand and soil and grown in a shaded greenhouse with natural sunlight during the day (maximum of 800 μmol m^−2^ s^−1^) and relative humidity of 65 % (±5 %). Mean daytime maximum and the minimum temperature in the greenhouse were 28 and 22 °C, respectively. Plants were fertilized once per week with a half-strength Hoagland solution. About 2 months later, selected uniform-sized seedlings were randomly separated into the several groups of 10 plants each for further experiment. Nine groups were placed into nine identical growth chambers with a 12-h photoperiod, photosynthetic flux density (PPFD) of 500 μmol m^−2^ s^−1^ at leaf height and constant relative humidity of ∼65 %. The chambers differed only in temperature. Three chambers were kept at 28 °C throughout the experimental period as non-stressed control, three chambers were kept at 35 °C as moderate HT stresses and the others were kept at 40 °C as severe HT stresses. In the HT growth chambers, water was regularly supplied during the day to prevent drought stress. At the beginning of the experiment (0 h), half of the control or HT-stressed plants in each chamber were treated with 24-epibrassinolide (EBR) at 0.25 μM at the rate of 15 mL per plant and the other half was simultaneously sprayed with distilled water containing same ratio of ethanol. In all cases, EBR were first dissolved in ethanol at concentrations of 0.2 mM, and then diluted to the designated concentrations with distilled water. The EBR concentration was selected on the basis of our preliminary experiments (data not shown). After 48 h of HT stress treatment, the leaves were harvested from the seedlings of each treatment for estimations of various physiological and biochemical parameters. Throughout the experiment, all the measurements were performed on the second fully expanded leaf from the top for each treatment. Each treatment was replicated at least three times under the same conditions.

### Determination of chlorophyll content and relative water contents

Chlorophyll concentration was determined according to the methods of Hasanuzzaman *et al*. (2012*b*). For determination of relative water content (RWC), cut leaves were weighed to determine fresh weights (FW). Leaves were then placed in distilled water, and turgid weights (TW) were measured after the leaf becoming fully turgid. Dry weights (DW) were measured after oven drying at 80 °C for 60 h. Relative water content was calculated using the equation:

$$\text{RWC}\;(\%)=100\times \frac{\text{FW}-\text{DW}}{\text{TW}-\text{DW}}.$$

### Extraction and measurement of ascorbate and glutathione

Leaf tissue (0.5 g) was ground in liquid nitrogen and homogenized in 3.5 mL cooled 5 % (w/v) metaphosphoric acid containing 1 mM EDTA at 4 °C using a mortar and pestle. The homogenate was centrifuged at 12 000*g* for 15 min at 4 °C, and the supernatant was collected for analysis of AsA and GSH. Ascorbate and DHA levels in the supernatant were determined according to Jiang *et al*. (2013). The glutathione pool was assayed according to previously described methods (Hasanuzzaman *et al*. 2011*a*). The content of GSH was calculated by subtracting oxidized glutathione (GSSG) from total GSH.

### Enzyme extraction and activity determination

About 0.50 g of frozen leaves were ground to a powder using a chilled mortar and pestle with liquid nitrogen and a small amount of insoluble polyvinylpolypyrrolidone, then homogenized with 3.5 mL cooled extraction buffer containing 50 mM K-phosphate (pH 7.5), 1 mM EDTA, 10 mM MgCl_2_, 1 mM ascorbic acid, 12 % (v/v) glycerol and 0.1 % (v/v) β-mercaptoethanol. Samples assayed for SOD and CAT activities were extracted in the same buffer without the ascorbic acid. The homogenates were centrifuged at 12 000*g* for 10 min and the supernatants were used for determination of enzyme activity. All enzyme extractions and centrifugations were carried out at 4 °C. The enzyme activity of each sample was measured four times at 25 °C. The total soluble protein (TSP) content was determined with the dye-binding method introduced by Bradford (1976) using bovine serum albumin (BSA) as a standard. Activities of SOD (EC 1.15.1.1) and CAT (EC: 1.11.1.6) were assayed with the methods previously described by Verma and Mishra (2005). The activity of APX (EC 1.11.1.11), MDHAR (EC 1.6.5.4), DHAR (EC 1.8.5.1) and GR (EC 1.6.4.2) were tested according to Hasanuzzaman *et al*. (2011*a*). Glutathione *S*-transferase (EC: 2.5.1.18) activity was estimated with 1-chloro-2,4-dinitrobenzene (CDNB) substrate as was described by Urbanek *et al*. (2005) with some modifications. The concentrations of reagents in 3 mL were: 100 mM Tris–HCl buffer (pH 6.5), 2 mM GSH, 1 mM CDNB and 200 μL enzyme extract. The enzyme reaction was initiated by the addition of CDNB and the increase in absorbance was measured at 340 nm for 2 min. Glutathione peroxidase (EC 1.11.1.9) activity was followed by the decrease in A340, resulting from NADPH oxidation (Elia *et al*. 2003).

Gly I (EC: 4.4.1.5) activity was assayed according to Upadhyaya *et al*. (2011) with some modifications. The concentrations of reagents in 3 mL were: 100 mM K-phosphate buffer (pH 7.0), 15 mM MgSO_4_, 1.7 mM GSH and 3.5 mM MG and 200 μL enzyme extract. The reaction was initiated by the addition of MG and the increase in absorbance was recorded at 240 nm. The determination of Gly II (EC: 3.1.2.6) activity was done by monitoring the decrease in the absorbance at 240 nm due to the hydrolysis of the substrate, *S*-d-lactoylglutathione (Saxena *et al*. 2005). The specific activity for glyoxalases I and II is expressed in units per milligram of protein (U/mg protein).

### Determination of superoxide radical (O_2_^•−^) producing rate and H_2_O_2_ content

The production rate of ${{\text{O}}_{2}}^{∙-}$ was measured by monitoring the nitrite formation from hydroxylamine in the presence of ${{\text{O}}_{2}}^{∙-}$ following the method of Verma and Mishra (2005). The content of H_2_O_2_ was measured by monitoring the A415 titanium-hydro peroxide complex following the method described by He *et al*. (2006).

### Determination of lipid peroxidation and methylglyoxal content

Lipid peroxidation was determined by estimating the malondialdehyde (MDA) content according to the method of Dhindsa *et al*. (1981). Methylglyoxal (MG) level was determined according to the method described by Yadav *et al*. (2005*a*). The assay mixture of 1 mL contains 250 μL 7.2 mM 1,2-diaminobenzene, 100 μL 5 M perchloric acid and 650 μL sample extract. The absorbance was read at 336 nm. Final concentration of MG was calculated from standard curve of pure MG.

### Statistical methods

All statistical analyses were conducted using SYSTAT version 13 (SYSTAT Software Inc. 2009). Data were statistically analysed using ANOVA, and tested for significant (*P* < 0.05) treatment differences using Tukey's honestly significant difference (HSD) test.

## Results

As shown in Table 1, the RWC and chlorophyll content of *F. concinna* seedlings were decreased, and the chlorophyll *a*/*b* ratio was increased upon exposure to HT stress. At 40 °C, RWC and chlorophyll content were significantly decreased by 22.6 and 27.1 %, respectively, compared with control (28 °C). The 40 °C-treated seedlings sprayed with EBR significantly increased the RWC and chlorophyll content and decreased the chlorophyll *a*/*b* ratio compared with the seedlings exposed to heat alone. However, the control (28 °C) and 35 °C-treated seedlings sprayed with EBR showed no significant differences in RWC, chlorophyll content and chlorophyll *a*/*b* ratio compared with the seedlings not sprayed with EBR. The interaction of temperature × EBR significantly affected chlorophyll content and chlorophyll *a*/*b* ratio.

**Table 1.** Relative water content (RWC), total chlorophyll (Chl *a* + *b*) and ratio of chlorophyll *a* to *b* (Chl *a*/*b*) in *F. concinna* seedlings subjected to EBR (0.25 μM) and high-temperature treatments. *Ficus concinna* seedlings were grown for 48 h with three different temperatures with or without EBR pretreatment. Data are means and SE of 6–8 replicate plants per treatment. Values indicated with different letters are significantly different (*P* < 0.05).

Treatment	RWC (%)	Chl *a*+*b* (mg g^−1^ FW)	Chl *a*/*b*
28 °C	92.4 ± 4.2a	1.18 ± 0.04ab	2.43 ± 0.14c
35 °C	86.7 ± 6.3a	1.11 ± 0.03b	2.56 ± 0.07c
40 °C	71.5 ± 4.7b	0.86 ± 0.02d	3.23 ± 0.11a
28 °C + EBR	93.1 ± 3.4a	1.21 ± 0.04a	2.54 ± 0.08c
35 °C + EBR	91.6 ± 5.3a	1.17 ± 0.03ab	2.61 ± 0.12c
40 °C + EBR	84.5 ± 4.4a	1.02 ± 0.04c	2.92 ± 0.09b

**Figure 1.**
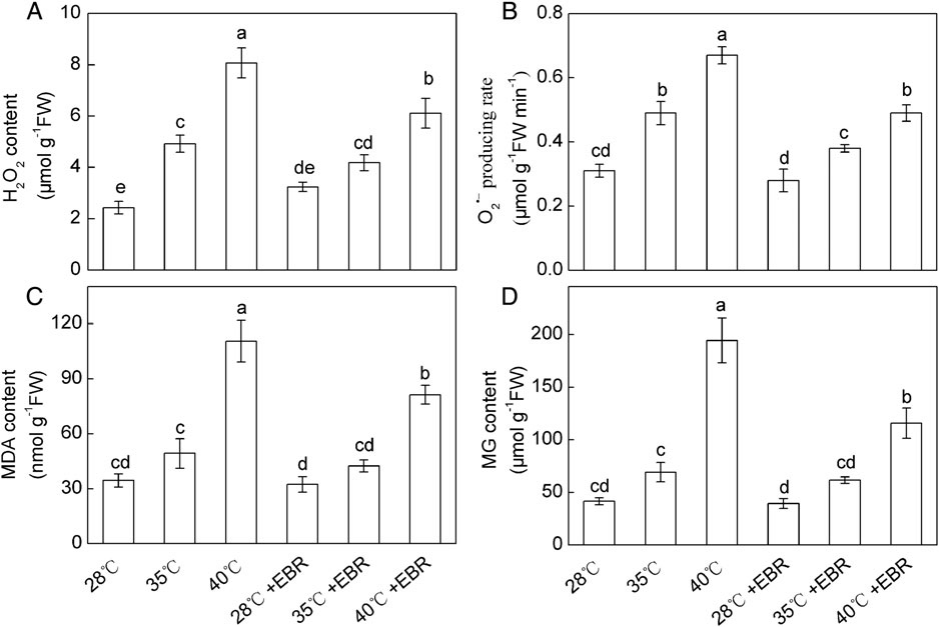
Hydrogen peroxide (H_2_O_2_) content (A), superoxide radical $({{\text{O}}_{2}}^{∙-})$ producing rate (B), malondialdehyde (MDA, C) and methylglyoxal (MG, D) content in *F. concinna* seedlings grown under three different temperatures with or without EBR pretreatment. All measurements were made at 48 h after treatment. Each bar represents the mean ± SE calculated from three independent experiments. Bars with different letters are significantly different at *P* < 0.05.

High temperature stress strongly increased the H_2_O_2_ content and ${{\text{O}}_{2}}^{∙-}$ production rate in *F. concinna* seedlings. Compared with the controls (28 °C), H_2_O_2_ content and ${{\text{O}}_{2}}^{∙-}$ production rate increased by 102.5 and 58.1 %, respectively, at 35 °C. When temperature was increased to 40 °C, H_2_O_2_ content and ${{\text{O}}_{2}}^{∙-}$ production rate increased 3.3- and 2.2-fold, respectively (Fig. 1A and B). Sharp increases in MDA and MG content were observed in the seedlings exposed to 40 °C treatment which were, respectively, 219.7 and 367.9% higher than that of control (Fig. 1C and D). The seedlings pretreated with EBR, when exposed to 40 °C treatment, significantly reduced the ${{\text{O}}_{2}}^{∙-}$ production rate and the levels of H_2_O_2_, MDA and MG, compared with HT only (Fig. 1C and D). However, at 28 and 35 °C, EBR-pretreated seedlings showed no significant change in the levels of H_2_O_2_, MDA and MG. Moreover, the H_2_O_2_, MDA and MG contents and ${{\text{O}}_{2}}^{∙-}$ production rate were all significantly affected by the interaction of temperature × EBR.

**Figure 2.**
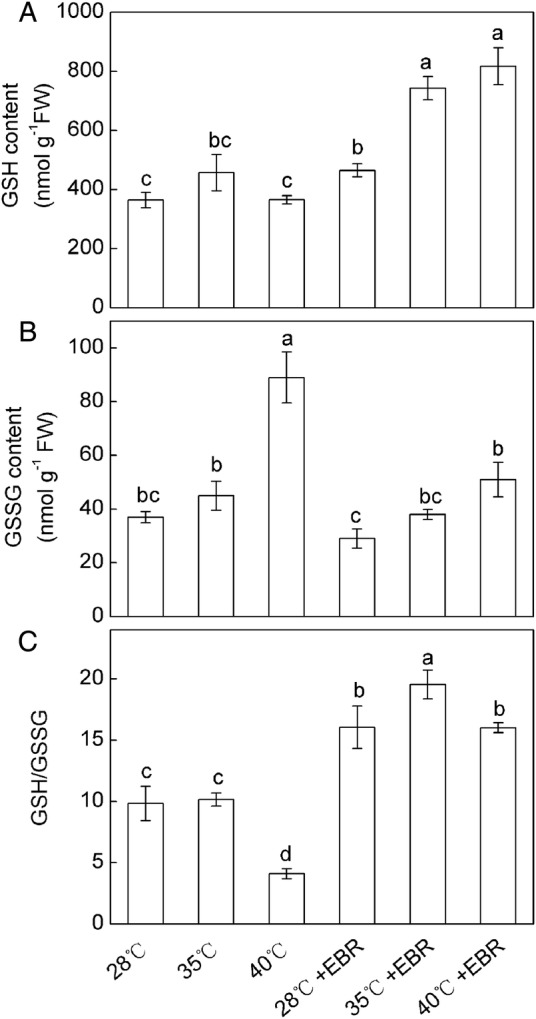
Glutathione accumulation in *F. concinna* seedlings grown under three different temperatures with or without EBR pretreatment. (A) Reduced glutathione (GSH), (B) oxidized glutathione (GSSG), and (C) GSH/GSSG ratio. All measurements were made at 48 h after treatment. Each bar represents the mean ± SE calculated from three independent experiments. Bars with different letters are significantly different at *P* < 0.05.

No significant difference in GSH content was observed in *F. concinna* seedlings exposed to HT compared with the control (Fig. 2A). Similarly, no significant differences in GSSG content and GSH/GSSG ratio were detected between controls and 35 °C-treated seedlings. A significant increase in GSSG content and a significant decrease in the GSH/GSSG ratio were apparent only under 40 °C treatments (Fig. 2B and C). Epibrassinolide-pretreatment showed a significant increase in the levels of GSH and GSH/GSSG at each temperature, and a significant decrease in the GSSG content at 40 °C than the levels in HT-treated seedlings without EBR. Moreover, the contents of GSH and GSSG and the ratio of GSH/GSSG were all significantly affected by the interaction of temperature × EBR.

**Figure 3.**
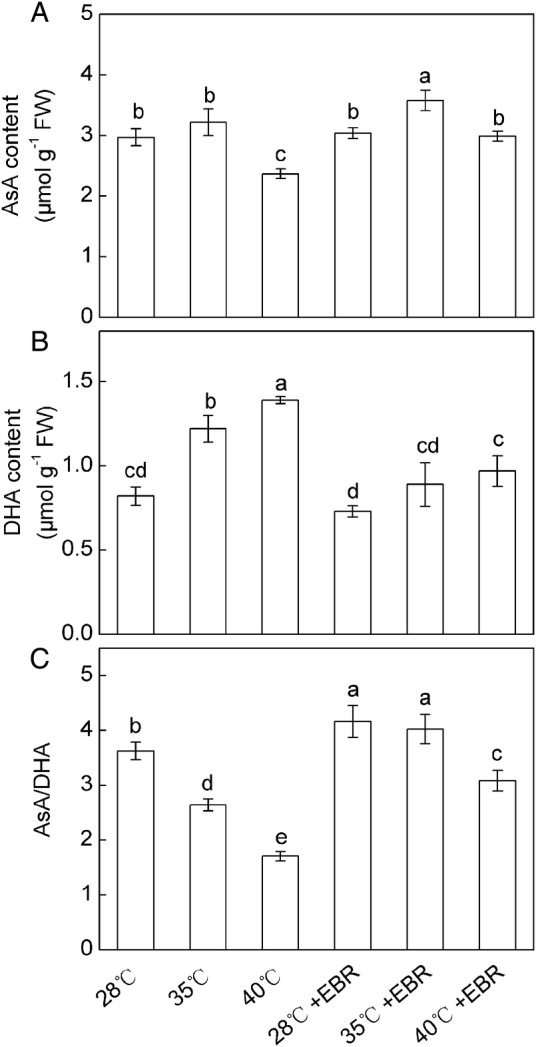
Redox states of ascorbate in *F. concinna* seedlings grown under three different temperatures with or without EBR pretreatment. (A) Reduced ascorbate (AsA), (B) oxidized ascorbate (DHA), (C) AsA/DHA ratio. All measurements were made at 48 h after treatment. Each bar represents the mean ± SE calculated from three independent experiments. Bars with different letters are significantly different at *P* < 0.05.

Ascorbate content in *F. concinna* seedlings has no significant change at 35 °C, whereas at 40 °C a significant decrease was observed compared with the control (Fig. 3A). The DHA content significantly increased by 48.8 and 69.5 %, and AsA/DHA ratio significantly decreased by 27.1 and 52.9 % in *F. concinna* seedlings of 35 and 40 °C treatments, respectively, compared with control values (Fig. 3B and C). However, the seedlings pretreated with EBR, when exposed to HT stress, significantly increased the AsA content and AsA/DHA ratio and decreased the level of DHA, compared with imposition of HT only (Fig. 3). Moreover, the contents of AsA and DHA and the ratio of AsA/DHA were all significantly affected by the interaction of temperature × EBR.

**Figure 4.**
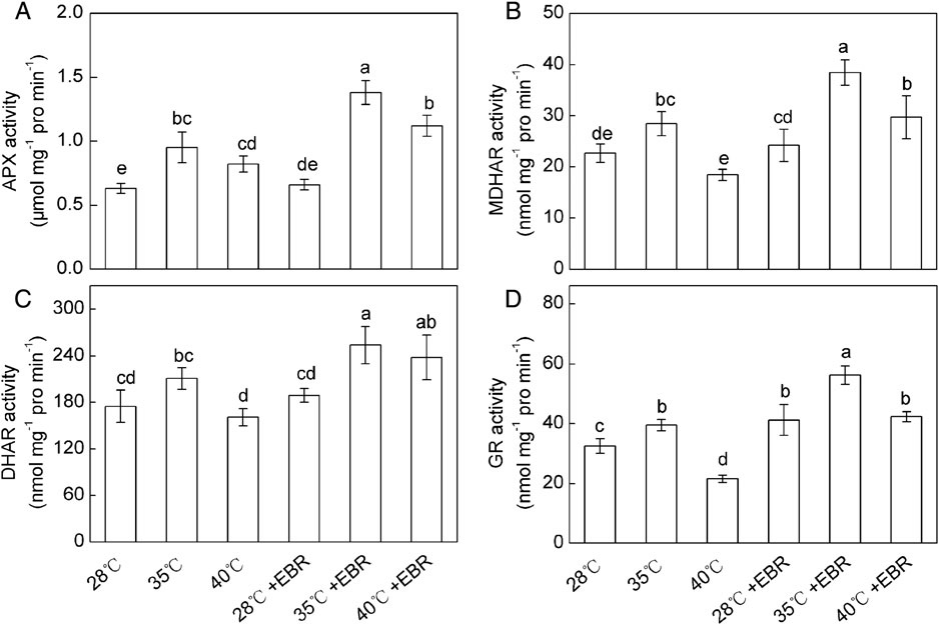
Activities of APX (A), MDHAR (B), DHAR (C) and GR (D) in *F. concinna* seedlings grown under three different temperatures with or without EBR pretreatment. All measurements were made at 48 h after treatment. Each bar represents the mean ± SE calculated from three independent experiments. Bars with different letters are significantly different at *P* < 0.05.

A significant increase in the APX activity was observed in response to HT stress (by 50.8 and 30.2 % at 35 and 40 °C treatments, respectively) compared with the control (Fig. 4A). The MDHAR, DHAR and GR activities in *F. concinna* seedlings were significantly increased at 35 °C compared with the control. However, at 40 °C, GR activity was significantly decreased (33.6 %), and no significant changes in MDHAR and DHAR activities were observed compared with the control (Fig. 4B–D). The EBR-pretreated *F. concinna* seedlings had higher activities of APX, MDHAR, DHAR and GR and, particularly, a significant increase was observed in the HT-treated plants, compared with the seedlings without EBR pretreatment (Fig. 4). Moreover, the interaction of temperature × EBR significantly affected the activities of APX, MDHAR, DHAR and GR.

**Figure 5.**
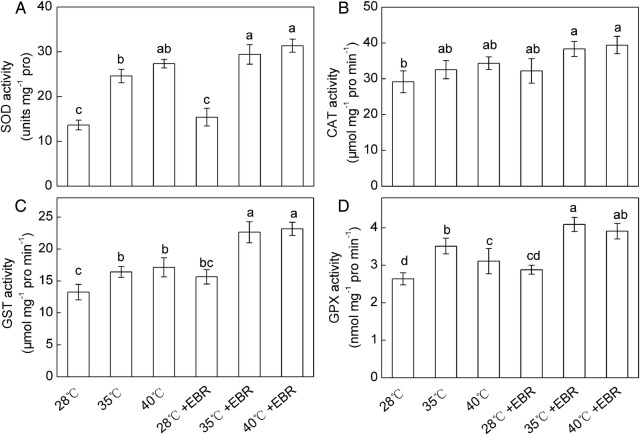
Activities of SOD (A), CAT (B), GST (C) and GPX (D) in *F. concinna* seedlings grown under three different temperatures with or without EBR pretreatment. All measurements were made at 48 h after treatment. Each bar represents the mean ± SE calculated from three independent experiments. Bars with different letters are significantly different at *P* < 0.05.

Significant increases in SOD, GST and GPX activities were observed in response to HT stress, compared with the control. The EBR-pretreated seedlings showed further increases in SOD, GST and GPX activities compared with seedlings without EBR treatment (Fig. 5A, C and D). A slight increase in CAT activity was observed under HT stresses, compared with the control, but this increase is not statistically significant. Similarly, EBR-pretreated HT-stressed seedlings showed slight increase in CAT activity compared with seedlings subjected to HT stress without EBR pretreatment (Fig. 5B). Moreover, the interaction of temperature × EBR significantly affected the GST activity.

**Figure 6.**
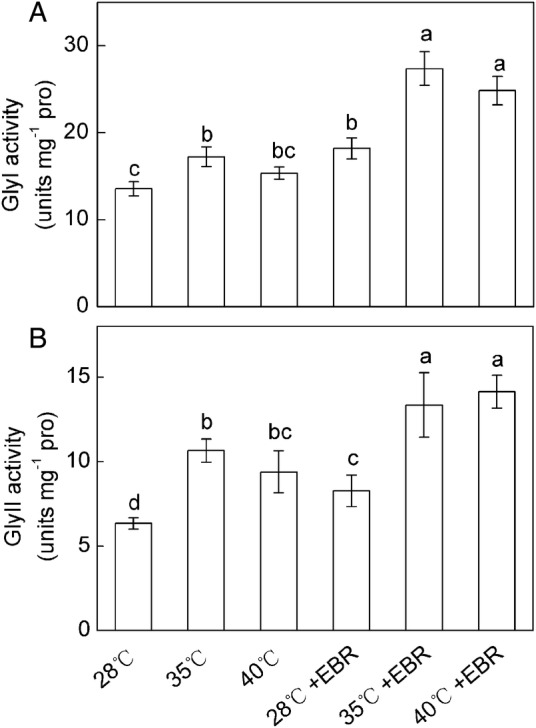
Activities of GlyI (A) and GlyII (B) in *F. concinna* seedlings grown under three different temperatures with or without EBR pretreatment. All measurements were made at 48 h after treatment. Each bar represents the mean ± SE calculated from three independent experiments. Bars with different letters are significantly different at *P* < 0.05.

A significant increase (27.1 %) in GlyI activity was observed at 35 °C but not at 40 °C, compared with the control (Fig. 6A). Significant increases in GlyII activity were observed (67.9 and 48.1 % at 35 and 40 °C, respectively) in response to HT stress, compared with the control (Fig. 6B). The EBR pretreated plants showed a significant increase in activities of GlyI and GlyII compared with the plants without EBR pretreatment (Fig. 6A and B). Moreover, the interaction of temperature × EBR significantly affected the activities of GlyI and GlyII.

## Discussion

Decreases in chlorophyll content and disruptions in plant water-balance are general consequences of HT stress. As expected, we found that HT induced declines in chlorophyll and RWC (Table 1), indicating that HT exerted its injury by inhibiting chlorophyll biosynthesis and generating osmotic stress. These effects were reverted by EBR-pretreatment, suggesting the protecting role of EBR against heat injury (Wu *et al*. 2014). It has been previously reported that HT may induce oxidative stress and ROS generations (Jin *et al*. 2010; Suleman *et al*. 2012). Lipid peroxidation is an oxidative stress marker used to indicate membrane damage. Reactive oxygen species cause the autocatalytic peroxidation of membrane lipids and pigments thus leading to the loss of membrane semi-permeability and modifying its functions (Xu *et al*. 2006). Although ROS initiate several oxidatively destructive processes, they also trigger various signalling pathways, and maintenance of appropriate ROS levels might represent a survival response (Apel and Hirt 2004; Suzuki and Mittler 2006). Reactive oxygen species at low concentrations are suggested to be involved in the signalling of many processes; however, excessive accumulation of ROS is one of the indicators of oxidative stress (Bhattacharjee 2005; Suzuki and Mittler 2006). In this study, HT significantly increased the H_2_O_2_ and content (Fig. 1). However, the magnitude of increase in MDA and MG content was much less than that in H_2_O_2_ in 35 °C-treated plants, which indicated that the increase in H_2_O_2_ content in 35 °C-treated plants might play a key role in mediating important signal transduction events, activating stress-response pathways and inducing defence mechanisms. On the other hand, the higher levels of MDA, ${{\text{O}}_{2}}^{∙-}$ and H_2_O_2_ observed in 40 °C-treated plants indicated that extreme HT induced severe oxidative ${{\text{O}}_{2}}^{∙-}$ stress and that the defence system by antioxidant enzymes was insufficient to protect the plants. Our results showed that EBR pretreatment followed by HT stresses displayed attenuated thermal injury coupled with lower MG and MDA content and less accumulation of ${{\text{O}}_{2}}^{∙-}$ and H_2_O_2_ at 40 °C, which indicated that exogenous EBR has a key role in ROS scavenging and reduction of oxidative stress in HT-stressed *F. concinna* seedlings. Similar protective effects of BRs in different herbaceous plants were observed under HT (Mazorra *et al*. 2002; Ogweno *et al*. 2008), cold (Jiang *et al*. 2013) and salinity (Ali *et al*. 2008; Hayat *et al*. 2010) stress conditions.

Brassinosteroids can interact with ROS in various ways, inhibiting lipid peroxidation and serving an antioxidant function including enzymatic and non-enzymatic systems during various stresses (Bajguz and Hayat 2009). AsA can directly scavenge ${{\text{O}}_{2}}^{∙-},$ H_2_O_2_, ^1^O_2_ and •OH (Noctor and Foyer 1998; Karuppanapandian *et al*. 2011). Glutathione is also regarded as an integral part of antioxidative system of the plant cell against oxidative stress, and contributed to the cellular defence and protection (Potters *et al*. 2002). AsA and GSH are important antioxidants in plants, and high levels of both AsA and GSH pools are a requisite for AsA–GSH cycle (Foyer and Shigeoka 2011). AsA–GSH cycle plays an important role in keeping the equilibrium between the ROS production and scavenging. The cycle includes APX, MDHAR, DHAR and GR that are involved in detoxification of H_2_O_2_ in a series of cyclic reactions (Noctor and Foyer 1998). In this study, the activities of APX, MDHAR, DHAR and GR were increased at 35 °C (Fig. 4), which suggested that under such condition the activities of AsA–GSH cycle enzymes could efficiently regenerated AsA and GSH. The maintained AsA and GSH may eliminate overproduced ROS as observed by lower levels of ${{\text{O}}_{2}}^{∙-},$ thereby resulted in the lower levels of MDA at 35 °C. However, at 40 °C, the activities of DHAR and GR were decreased, suggesting that the AsA–GSH cycle was interrupted under severe HT stress. Our findings support those of Mostofa *et al*. (2014) who reported that the AsA–GSH cycle was highly sensitive to severe heat stress. Our results also showed that severe HT (40 °C) led to substantial declines in AsA and AsA/DHA ratio (Fig. 3), which might be one of the ways inducing HT injury probably due to increased oxidation for scavenging overproduced ROS and inefficient AsA–GSH cycle. Additionally, the increased APX activity also results in decreasing in AsA content (Fig. 4). However, amendment of this trend by EBR pretreatment might restrict the membrane damage and prevent oxidative injury, and ultimately shepherd to a better antioxidant capacity against HT. Epibrassinolide might take part in the regeneration of AsA by upregulating the activities of MDHAR and DHAR (Fig. 4). The increase in AsA levels can be demanded for the increased APX activity in order to control the increase in H_2_O_2_ generated by the HT stress. Epibrassinolide-pretreatment also enhanced activities of AsA–GSH cycle enzymes and AsA content in cucumber under cold stress (Jiang *et al*. 2013). Additionally GSH/GSSG ratio plays a drastic role in the maintenance of the cellular homeostasis and signalling system in plants (Jiang *et al*. 2012, 2013), and the ratio also may be involved in ROS perception (Shao *et al*. 2008). Our findings suggested that higher ROS content under 40 °C HT stress might contribute to the oxidation of GSH to GSSG and concomitant decrease of GSH/GSSG ratio (Fig. 2). On the other hand, GSH is linked to the detoxification of H_2_O_2_ via GR in the AsA–GSH cycle and organic peroxides via GPX (Paradiso *et al*. 2008; Hasanuzzaman and Fujita 2013). Therefore, the less increase of GSH in 40 °C-treated leaves may be due to decreased activities of GR and/or the reaction of GSH with oxyradicals generated due to oxidative stress. The GSSG content was largely increased while the DHAR activity was slightly decreased or remained unchanged at 40 °C, which indicated that the increased GSSG content in 40 °C-treated plant may be due to the reaction of GSH with oxyradicals generated by oxidative stress or decreased GR activity (Aravind and Prasad 2005; Hasanuzzaman *et al*. 2011*a*). However, corresponding with GR activity, HT-stressed or non-stressed plants which were pretreated with EBR, showed a higher increase in GSH content and GSH/GSSG ratio than did the plants grown without EBR pretreatment. These results provide a clear demonstration of the role of EBR in both regulation redox regulation and GSH biosynthesis or regeneration. Enhanced GSH contents in EBR pretreatment plants may partly account for the higher capacity for oxidative defence during HT stress.

The improvement in stress tolerance is also associated with the antioxidant system in plants. Superoxide dismutase, CAT, GPX and GST are the most important antioxidant enzymes in plants. Our results showed that the HT-stressed *F. concinna* seedlings fought against ROS production by raising the activities of SOD, CAT, GPX and GST (Fig. 5), and that the increase in SOD activity correlated with the increase in H_2_O_2_ content. However, at 40 °C, the increased SOD, CAT, GPX and GST activities in HT-stressed seedlings might not be sufficient to deal with excess H_2_O_2_ and ${{\text{O}}_{2}}^{∙-}$ since the AsA–GSH cycle was depressed under the condition. Our results support the finding of Hasanuzzaman *et al*. (2012*b*) who observed significant increase in GPX and GST activities under heat stress. Glutathione peroxidases are a family of isozymes that use GSH to reduce H_2_O_2_ and lipid hydroperoxides (LOOHs), and therefore protect plant cells from oxidative stress (Noctor *et al*. 2002). Glutathione *S*-transferases are another important antioxidative component often involved in nucleophilic conjugation of GSH to a wide variety of organic molecules (Dixon *et al*. 2010). Therefore, the enhancement of GPX and GST activities may be two important reasons of the less increase of GSH content under severe HT stress. In contrast, Mostofa *et al*. (2014) reported that the activities of GPX and GST in the rice seedlings during heat stress correlated with the fluctuation in GSH level. Glutathione *S*-transferases can also eliminate H_2_O_2_ and electrophilic xenobiotics (Noctor *et al*. 2012). Therefore, EBR-pretreated HT-stressed seedlings significantly enhanced the activities of SOD, GPX and GST, and slightly increased the CAT activity and thus contributed to the reduction of H_2_O_2_, ${{\text{O}}_{2}}^{∙-}$ and MDA levels under HT stress, and subsequently affirmed higher tolerance to HT stress. Glutathione reductase-mediated regeneration of GSH in leaves of EBR-treatment seedlings may contribute to substrates for GPX and GST. This effect may represent the antioxidant property of EBR for suppressing the high levels of HT-triggered ROS.

Methylglyoxal is a potent cytotoxic compound produced spontaneously from the glycolysis pathway under abiotic stresses (Yadav *et al*. 2005*a*). An increase in MG level in plant cells further intensifies the production of ROS by inactivating the antioxidant enzymes (Hoque *et al*. 2012). Therefore, detoxification of this compound is also a priority to register higher tolerance to oxidative stress. Higher Gly I and Gly II activities could protect plants against MG accumulation during abiotic stresses (Jain *et al*. 2002; Hossain and Fujita 2009, 2010; Hasanuzzaman *et al*. 2011*b*). In this study, MG content increased under HT stress conditions despite the increased activities of Gly I and Gly II in HT-treated seedlings compared with the control plants (Fig. 6), suggesting that the detoxification of MG via the glyoxalase system was not sufficient. Additionally, HT-treated seedlings supplemented with EBR further increased the activities of Gly I and Gly II. This enhanced activities of glyoxalase system decreased the MG content in *F. concinna* seedlings under HT stress condition. Our work is the first study to illustrate the role of EBR in maintaining the higher glyoxalase activity and resulting in decrease in MG content. High Gly I and Gly II activities also create the possibility of upregulating the GSH level and the GSH/GSSG ratio via the glyoxalase system (Hasanuzzaman *et al*. 2011*b*; Hasanuzzaman and Fujita 2013).

## Conclusions

In conclusion, the oxidative damage was occurred in *F. concinna* seedlings under HT stress, as indicated by the higher levels of ${{\text{O}}_{2}}^{∙-},$ H_2_O_2_, MDA and MG, and the lower levels of RWC and chlorophyll content, likely due to inefficient induction of non-enzymatic and enzymatic antioxidants as well as of the glyoxalase systems. On the other hand, our results with *F. concinna* under HT stress clearly showed that EBR treatment reduced HT-induced increases in ${{\text{O}}_{2}}^{∙-},$ H_2_O_2_ and MG levels and this process is likely associated with a decrease in lipid peroxidation. Epibrassinolide attenuated the HT-induced oxidative stress by simultaneously increasing non-enzymatic and enzymatic antioxidant responses, as well as MG detoxification systems. However, a complete elucidation of the regulatory role of endogenous EBR, as well as its detailed signalling mechanism, would be helpful in improving our understanding of EBR-mediated HT stress tolerance.

## Sources of Funding

The work was partly supported by the National Natural Science Foundation of China (nos 31170584, 31200525 and 30901144) and the Zhejiang Provincial Natural Science Foundation of China (nos Y3090276 and Y3110286).

## Contributions by the Authors

S.H.J. planned the work and wrote the paper. S.H.J. and X.Q.L. were responsible for the experimental design and carried out the experiments. G.G.W. analysed the data and edited the manuscript. X.T.Z. assisted in the performance of the experiments.

## Conflicts of Interest Statement

None declared.
